# THE CENTRALITY OF GENDER EXPRESSION FOR TRANSGENDER ELDERS AND CONCEPTUALIZATIONS OF SUCCESSFUL AGING

**DOI:** 10.1093/geroni/igz038.2700

**Published:** 2019-11-08

**Authors:** Steffany Sloan, Jacquelyn Benson

**Affiliations:** University of Missouri-Columbia, Columbia, Missouri, United States

## Abstract

Successful aging is a construct regularly addressed in the gerontological literature, most typically referred to as living in the best physical and cognitive health possible, experiencing the least amount of disease, and engaging actively within one’s social environment. There are, however, conceptually distinct aspects of aging for older adults who identify as transgender, particularly given lifelong experiences of marginalization and stigma. In order to identify factors transgender older adults consider most relevant to successful aging, a Theory-Generating Qualitative Meta-synthesis was conducted. The study utilized systematic methods and thematic analysis with qualitative data from empirical studies focusing on transgender aging experiences, particularly drawing upon data that amplified the transgender older adult point of view. Findings of this study indicate that although trans older adults do not minimize unique challenges inherent to aging as a gender minority, successful aging rests on the relief and resilience offered by authentic gender expression. As such, data from this study informed the development of a model of successful aging that centralizes the importance of gender expression. Understanding the central role of gender expression in later life can be especially impactful for social and health service providers, as it emphasizes gender affirming experiences as highly salient to one’s own assessment of quality aging. Furthermore, conceptualizing aging for transgender elders in a way that prioritizes the importance of identity can aid in preventing an unnecessarily deficits-based perspective for service providers, and supports the necessity of amplifying a culturally specific understanding of successful aging.

